# Boosting the Brain Delivery of Atazanavir through Nanostructured Lipid Carrier-Based Approach for Mitigating NeuroAIDS

**DOI:** 10.3390/pharmaceutics12111059

**Published:** 2020-11-06

**Authors:** Saif Ahmad Khan, Saleha Rehman, Bushra Nabi, Ashif Iqubal, Nida Nehal, Usama A. Fahmy, Sabna Kotta, Sanjula Baboota, Shadab Md, Javed Ali

**Affiliations:** 1 Department of Pharmaceutics, School of Pharmaceutical Education and Research, Jamia Hamdard, New Delhi 110062, India; iamsaif1994@gmail.com (S.A.K.); saleharehman90@gmail.com (S.R.); nabibushra79@gmail.com (B.N.); nidanehal123@gmail.com (N.N.); sbaboota@jamiahamdard.ac.in (S.B.); 2 Department of Pharmacology, School of Pharmaceutical Education and Research, Jamia Hamdard, New Delhi 110062, India; asifiqubal2013@gmail.com; 3 Department of Pharmaceutics, Faculty of Pharmacy, King Abdulaziz University, Jeddah 21589, Saudi Arabia; uahmedkauedu.sa@kau.edu.sa (U.A.F.); skotta@kau.edu.sa (S.K.); shaque@kau.edu.sa (S.M.); 4 Center of Excellence for Drug Research & Pharmaceutical Industries, King Abdulaziz University, Jeddah 21589, Saudi Arabia

**Keywords:** atazanavir, nanostructured lipid carriers, neuroAIDS, brain targeting, histopathology

## Abstract

Atazanavir (ATZ) presents poor brain availability when administered orally, which poses a major hurdle in its use as an effective therapy for the management of NeuroAIDS. The utilization of nanostructured lipid carriers (NLCs) in conjunction with the premeditated use of excipients can be a potential approach for overcoming the limited ATZ brain delivery. Methods: ATZ-loaded NLC was formulated using the quality by design-enabled approach and further optimized by employing the Box–Behnken design. The optimized nanoformulation was then characterized for several in vitro and in vivo assessments. Results: The optimized NLC showed small particle size of 227.6 ± 5.4 nm, high entrapment efficiency (71.09% ± 5.84%) and high drug loading capacity (8.12% ± 2.7%). The release pattern was observed to be biphasic exhibiting fast release (60%) during the initial 2 h, then trailed by the sustained release. ATZ-NLC demonstrated a 2.36-fold increase in the cumulative drug permeated across the rat intestine as compared to suspension. Pharmacokinetic studies revealed 2.75-folds greater C_max_ in the brain and 4-fold improvement in brain bioavailability signifying the superiority of NLC formulation over drug suspension. Conclusion: Thus, NLC could be a promising avenue for encapsulating hydrophobic drugs and delivering it to their target site. The results suggested that increase in bioavailability and brain-targeted delivery by NLC, in all plausibility, help in improving the therapeutic prospects of atazanavir.

## 1. Introduction

Acquired immunodeficiency syndrome (AIDS), a pandemic and perhaps the deadliest malady has claimed more than 39 million lives up until the year 2014. Currently, 37.3 million people have active HIV bringing about yearly demise of around 1.2 million individuals [1]. As per the World Health Organization (WHO), nearly 1.8 million people became newly infected and 9.4 million people died in 2017 due to AIDS. Further, as per the UNAIDS (The Joint United Nations Program on HIV/AIDS) data, India in 2017 witnessed 2.1 million individuals affected with AIDS, 69,000 deaths, 88,000 new HIV infections, 0.2% adult HIV prevalence, and 56% adults and 33% children were on antiretroviral therapy [2]. Thus, being a fatal disease of pandemic extents with continually rising global burden and distressing health-related and socioeconomic effects, AIDS has become a serious cause of concern worldwide.

The causative organism of AIDS is the human immunodeficiency virus (HIV), which debilitates the immune system by triggering the radical loss and dysregulation of the macrophages and CD4+T lymphocytes [3]. The mortality and morbidity rate linked to AIDS has shown a dramatic depreciation with the dawn of highly active antiretroviral therapy (HAART) [4]. The chronic administration of HAART, which is a combination of antiretroviral (ARV) drugs, proved quite effective in diminishing the viral burden and controlling its replication and transmission thereby assuring a long and productive life to the affected patients. However, the therapy lacked in offering a complete annihilation of the virus from the body owing to the existence of HIV in the anatomical reservoirs like the central nervous system (CNS), lymphatic system, lung, and liver. The virus in these reservoirs becomes latent and the presence of anatomical barriers limit the access to ARVs [5,6]. The ineptitude of the systemically administered ARVs to traverse the blood–brain barrier (BBB) makes the brain one of the predominant HIV reservoirs in the affected patients [7].

NeuroAIDS is consequently turning into a very common complication causing adverse neurological dysfunctions in the HIV-tainted patients. HIV primarily targets microglia, mononuclear macrophages and perivascular macrophages in the brain. The virus enters the brain via infected circulating monocytes and replicates itself thereby prompting reinfection and inducing the drug resistance [8]. It is realized that the vast majority of the currently available ARVs are unable to cross the impregnable BBB while some possessing this ability exhibit deficient concentration in the brain leading to their therapeutic failure [9]. Some of the other impediments in the path of ARVs moving to the brain include the absence of ideal physicochemical properties of ARVs, existence of efflux transporters like P-glycoprotein (P-gp) and metabolizing enzymes on BBB [5]. This entail administration of high doses, which is certainly not a practical solution as it prompts serious side effects like hematological intolerance when administered for a longer span [6]. This warrants identification of a suitable drug delivery system, which assures the successful delivery of ARVs into the brain for an effective management of AIDS.

Nanostructured lipid carriers (NLCs) appear to be a profound substitute for targeting ARVs into the brain. These lipid nanoparticles are known to experience lymphatic absorption, which ensures prolonged blood circulation of the ARVs and effective brain targeting. This will likewise guarantee the accessibility of the drugs at the viral sanctuaries (lymphatic system) and also avoid first-pass hepatic metabolism [10]. In the recent years, NLCs have encountered considerable attention for brain targeting, which can be attributed to the rapid brain uptake, inhibition of P-gp-mediated drug efflux, controlled and sustained drug release, presence of physiological lipids, biocompatibility, biodegradability, long-term storage stability, and less toxicity [11]. Ease of preparation, cost effectiveness, and industrial scalability are other advantages of NLC. Additionally, the presence of both liquid and solid lipid in the NLC steers to high drug entrapment and improved drug loading, which makes these carriers a promising contender for delivery of therapeutics past the brain [12].

The lipid digestion of NLC by the pancreatic enzymes present in the duodenum leads to the formation of monoglycerides and fatty acids, which further form micelles after being enveloped by bile salts. After these micelles reach the enterocytes, triglycerides are formed in the intestinal cells. On aggregation with phospholipids and cholesterol, these lipid constituents form chylomicrons, which are unable to cross the blood capillaries due to their large size [13]. The chylomicrons undergo lymphatic uptake, followed by the glymphatic system of the brain, where receptor mediated transcytosis (RMT) is the mechanism utilized by NLC [14,15]. After administration of nanoparticles, their concentration in the brain depends on factors like plasma-protein binding, permeability across BBB, efflux by efflux transporters, enzymatic metabolism, and plasma–concentration time curve [16]. Further, the endothelial cells bear a negative surface charge, which is a crucial component of the defense system of BBB, as it regulates the permeation of the positively charged molecules across BBB [17]. Atazanavir (ATZ), an azapeptide derivative is a protease inhibitor utilized for the treatment therapy of HIV. It acts selectively on HIV-1, attaching to the protease active site of the HIV-1 protease enzyme and inhibiting its activity [18]. ATZ is a BCS class II drug with low aqueous solubility (0.003 mg/mL) and high permeability (log P 4.11). The drug suffers from extensive hepatic metabolism, and undergoes an efflux by P-gp efflux transporters, consequently leading to low oral bioavailability and lesser brain biodistribution. Moreover, traversing through the BBB becomes more challenging owing to the extensive plasma protein binding and high molecular weight [19]. Although a solid lipid nanoparticle (SLN) and self-nanoemulsifying drug delivery system (SNEDDS) of ATZ for brain targeting have been prepared earlier, none of the research report formulation of ATZ-loaded NLC [19,20]. NLCs are reported to overcome the several disadvantages of SLN like poor drug loading (due to the absence of liquid lipid) and drug expulsion on prolonged storage. Moreover, in SLN, only up to 30% lipid can be used as beyond 30%, it leads to the formation of bicoherent systems. For NLC, the lipid ratio allowed in the formulation is up to 95%, which is much higher than SLN. Based on these disadvantages, the application of SLN is limited and NLCs are more favorable delivery system [9,21]. Further, SNEDDS of ATZ has also been prepared, but limitations like gastrointestinal irritation due to large amount of surfactants, precipitation of the drug in vivo, and instability due to variation in pH and temperature hinders its wide applicability [22,23]. NLC are devoid of such disadvantages, and thus forms the basis of this research

Accordingly, in the present investigation, the potential of NLCs was examined for augmenting the oral bioavailability and brain distribution of ATZ following its oral administration. The formulation was prepared using the quality by design (QbD)-based approach, which helps in examining all the factors included in formulating NLC and the interactions among such factors, so that a high quality formulation is obtained. The prepared formulation was subjected to in vitro and in vivo evaluations. The formulated NLCs were analyzed for brain targeting efficiency in Wistar rats. The findings suggested NLC to be a prospective carrier for the effective management of AIDS.

## 2. Materials and Methods

### 2.1. Materials

Atazanavir was obtained as a generous gift from Sun Pharmaceutical Industries Limited (Gurgaon, India). Labrafil^®^ M1944CS, Labrafil^®^ M2125CS, Labrafac^TM^ WL1349, Labrafac^TM^ PG, Lauroglycol^TM^ 90, Capryol^TM^ PGMC, Compritol^®^ 888ATO, Precirol^®^ ATO 5, Solutol HS15^®^, Glyceryl monostearate, Gelucire^®^ 44/14, and Gelucire^®^ 43/10 were acquired by Gattefosse (Saint Priest, France). Capmul^®^ PG-12 EP/NF, Captex^®^ 100, and Captex^®^ 300 were obtained from Abitech Corporations (Cleveland, OH, USA). The surfactants like span 20, tween 80, and tween 20 were procured from Merck (Hohenbrunn, Germany). Poloxamer 188, Poloxamer 407, and Cremophor RH 40 were acquired from BASF (Mumbai, India). Sesame oil, canola oil, and castor oil were acquired from the central drug house (Mumbai, India). Water, methanol, and acetonitrile for HPLC were procured from Fischer Scientific Co. (Mumbai, India). Purified water used for all experiments was obtained from Milli Q Plus (Millipore, MA, USA). Rest of the chemicals and reagents were procured from S.D. Fine Chemicals Ltd. (Mumbai, India). Freshly prepared buffer solutions were used for all experiments.

### 2.2. Animals Used

Adult albino Wistar rats of weight 200–250 g obtained from the Central Animal House, Jamia Hamdard, New Delhi, India were used. The animals were housed in cages kept under set laboratory specifications of 25 ± 2 °C temperature and 55% ± 5% relative humidity with a 12 h light–dark cycle. The animals had unrestricted access to standard diet and water. The investigation was carried out as per the protocols certified by Institutional Animal Ethics Committee of Jamia Hamdard, New Delhi, India (protocol approval number: 1519). The committee is listed in the Committee for the Purpose of Control and Supervision of Experiments on Animals (173/GO/Re/S/2000/CPCSEA).

### 2.3. Screening of Lipids and Surfactants

For selecting the liquid lipid, various lipids and oils like Captex^®^ 100, Captex^®^ 300, Capryol^TM^ PGMC, Capmul^®^ PG-12 EP/NF, Labrafac^TM^ WL1349, Labrafac^TM^ PG, and Lauroglycol^TM^ 90, Labrafil^®^ M1944CS, Labrafil^®^ M2125CS, castor oil, canola oil, and sesame oil were screened for solubility of ATZ. To each vial comprising 1 mL each of different liquid lipids, an excess quantity of ATZ was put in. The vials were equilibrated for 72 h in an isothermal shaker (25.0 ± 0.5 °C) and the centrifugation of these samples were done for 30 min at a speed of 5000 rpm by a high-speed centrifuge (Remi Instruments, Delhi, India). The supernatant was isolated, diluted in methanol, and analyzed at λ_max_ of 247 nm by the UV-spectrophotometer (UV 1700, Shimadzu, Japan). The lipids presenting highest solubility was chosen for preparing NLC [24].

The solid lipids (Gelucire^®^ 44/14, and Gelucire^®^ 43/10, Precirol^®^ ATO 5, Compritol^®^ 888ATO, and glyceryl monostearate) were added in small increments to the vials, each containing 10 mg of ATZ. The vials were heated at above 5 °C beyond the respective melting points of solid lipid. The amount of each solid lipid utilized for dissolving ATZ was determined. The lipid dissolving the drug completely in the minimum amount was chosen for NLC preparation. The end point was considered as the absence of any undissolved drug in the vials [10].

The liquid and solid lipids chosen for preparing NLC were blended in various proportions (9:1, 8:2, 7:3, and 6:4), melted and cooled down to room temperature. The blends were visually examined for uniformity, clarity, turbidity, and phase separation. The solid–liquid binary lipid (SLB) mixture showing good miscibility without any indication of phase separation or turbidity was selected for the design of NLC [25].

For selecting surfactants, 100 mg of SLB was placed in a glass vial and methylene chloride (3 mL) was put in to dissolve it. For each of the 5% solutions of various surfactants (Span 20, Poloxamer 188, Poloxamer 407, Cremophor RH 40, tween 80, and tween 20), 10 mL were prepared. Further, the SLB blend was put in the surfactant solution under magnetic stirring (Remi Instruments, Mumbai, India). The mixtures were kept at 40 °C to remove the organic phase and then diluted to 10-folds using Milli-Q water. UV-spectrophotometer was utilized for recording the percentage transmittance of each sample at 510 nm [26].

### 2.4. Quality by Design (QbD) Approach

**Figure 1:**
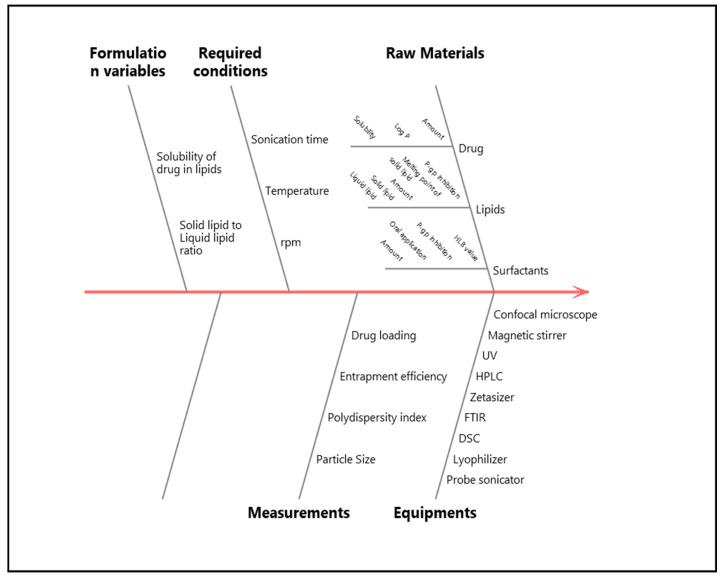
Ishikawa diagram for fabrication of atazanavir-nanostructured lipid carrier (ATZ-NLC).

The authors harnessed QbD for fabrication of ATZ-NLC so that a safe, effective, patient centric formulation evincing the desired characteristics can be produced. For this approach, quality target product profile (QTPP) was established. The process also involved identification of critical process parameters (CPPs) and critical material attributes (CMAs), which bear an influence on critical quality attributes (CQAs). Limits are defined for CQAs as they ascertain the final characteristics and quality of the formulation [27]. Table 1 exhibits the different aspects of QTPP and CQA while the risk assessment was established using the Ishikawa (fish-bone) diagram (Minitab 17 software, M/s Minitab Inc., Philadelphia, USA as shown in Figure 1.

### 2.5. Preparation of ATZ-NLC

ATZ-NLCs were developed by melt emulsification followed by the probe sonication method. The SLB containing Precirol ATO 5 (solid lipid) and Lauroglycol 90 (liquid lipid) in the ratio of 70:30 was melted at 70 °C. ATZ (3 mg) was then put into the melted SLB and allowed to dissolve. The aqueous phase contained Cremophor RH 40 (3% *w/v*) solubilized in 10 mL of water and kept at the same temperature. The hot aqueous mixture was then transferred gradually to the molten lipid phase and stirring was continued for 30 min at 500 rpm. This pre-emulsion was then treated for 5–15 min on an ice bath using a probe sonicator (Labsonic^®^ M) at 40% amplitude and 6 cycles to obtain NLC formulation of ATZ [28].

### 2.6. Optimizing ATZ-NLC by the Box–Behnken Design (BBD)

ATZ-NLC was optimized using response surface methodology (RSM), which quantifies the relationship amidst controllable independent variables and the responses obtained. A three-factor three-level BBD utilizing Design-Expert 11 software (Stat-Ease Inc., Minneapolis, MN, USA) was utilized to examine the effects of lipid concentration, surfactant concentration, and time of sonication on particle size, entrapment efficiency, and drug loading of NLCs. Table 2 represents the various levels (low, medium, and high) used for the independent and dependent variables. BBD yielded 17 experimental runs and a quadratic Equation (1), which is as given under:

(1) $$Y=b_{0}+b_{1}X_{1}+b_{2}X_{2\;}+\;b_{3}X_{3\;}+\;b_{12}X_{1}X_{2\;}+\;b_{13}X_{1}X_{3}\;+\;b_{23}X_{2}X_{3\;}+\;b_{11}X_{1}^{2}\;+\;b_{22}X_{2}^{2}\;+\;b_{33}X_{3}^{2}$$

where *Y* = measured response for each factor level combination, $b_{0}$ = intercept, $b_{1}-b_{3}$ = regression coefficients, $X_{1}\;-\;X_{3}$ = coded levels of independent variables, and $X_{i}X_{j}$ and $X_{i}^{2}$ (*i*, *j* = 1, 2 or 3) = interaction and quadratic coefficients of the observed experimental values [29].

### 2.7. Lyophilization of Optimized ATZ-NLC

The aqueous dispersions of ATZ-NLC was mixed with 1% *w*/*v* mannitol (cryoprotectant) and stored at −80 °C for 24 h. The freeze dryer (Metrex Scientific Instruments Pvt. Ltd., Delhi, India) was used for the lyophilization of the samples to obtain free-flowing dry powder for further characterization (like differential scanning calorimetry (DSC) and FTIR) [25].

### 2.8. Evaluation of Optimized ATZ-NLC

#### 2.8.1. Particle Size, Polydispersity Index (PDI), and Zeta Potential (ZP)

These parameters were estimated by Malvern Zetasizer (Nano-ZS; Malvern Instruments, Malvern, UK). Before the estimations, the samples were diluted using double distilled water (1:10) to yield an appropriate scattering intensity. ZP was measured using disposable polystyrene cells at 25 °C and at an angle of 90° [30].

#### 2.8.2. Drug Entrapment Efficiency (%EE) and Drug Loading Capacity (%LC)

The aqueous dispersions of ATZ-NLC were diluted with 0.1 N HCl to adjust its pH in the range of 1.5–2. This will cause aggregation of the NLCs thus making the separation easier during centrifugation. The samples were then centrifuged at 30,000 rpm for 30 min by means of a high-speed centrifuge (Sigma-3K30, Osterode am Harz, Germany). Isolation of the supernatant was done, suitably diluted with methanol, and analyzed via UV spectrophotometer. %EE and %LC are determined using the following Equations (2) and (3):

(2) $$\%\;Entrapment\;efficiency\;\left(\%\right)=\;\frac{W_{t\;}-\;W_{f}}{W_{t}}\;\times \;100\%$$

(3) $$\%\;Drug\;Loading\;\left(\%\right)=\;\frac{W_{t\;}-\;W_{f}}{W_{l}}\;\times \;100\%$$

where, *W_t_* = weight of initial drug added, *W_f_* = weight of unencapsulated drug present in supernatant, and *W_l_* = total weight of liquid lipid and solid lipid [25].

#### 2.8.3. Transmission Electron Microscopy (TEM)

ATZ-NLC was analyzed for particle size and surface morphology using TEM (CM 200, Philips Briarcliff Manor, NY, USA) operated at a voltage of 200 kV. A drop of aqueous dispersion of each sample (ATZ-NLC and placebo) was positioned on a carbon film-covered copper grid (400-mesh), which was followed by negative staining with 1% phosphotungstic acid. The excess liquid was removed, and the copper grid was dried out at room temperature. Later, the specimens were then analyzed under the microscope [31].

#### 2.8.4. Differential Scanning Calorimetry (DSC)

The DSC examination of ATZ and optimized ATZ-NLC was performed using DSC (Perkin Elmer, Pyris 6 DSC, USA) to understand their melting behavior and crystallinity. The instrument was operated from 30 to 400 °C, at nitrogen purging of 40 mL/min and heating rate of 10 °C/min. 1-2 mg of each samples was sealed in aluminum pans, equilibrated at 25 °C and exposed to DSC analysis. An empty sealed aluminum pan was treated as the reference [32].

### 2.9. In Vitro Release Study

The drug release from the suspension and optimized NLC of ATZ was assessed in 0.1 N HCl pH 1.2, phosphate buffer saline (PBS) pH 6.8 and PBS pH 7.4 (simulating CSF) using the dialysis technique. Typically, 3 mL each of drug suspension and ATZ-NLC, corresponding to 100 mg ATZ was put separately in activated dialysis bags (mol. wt. 12,000–14,000 Da; Hi Media, Mumbai, India). The bags were secured at both the ends and were then suspended in 100 mL of each media kept at 37 ± 0.5 °C and stirred at 50 rpm. About 2 mL of samples were drawn from each media at definite time intervals (0.25, 0.5, 1, 2, 4, 8, 12, and 24 h) and substituted with fresh dissolution medium at that same time-point to maintain sink conditions. The drug content was estimated for the withdrawn samples utilizing UV spectrophotometer at λ_max_ of 247 nm. The results were incorporated to various kinetic models like zero order, first order, Higuchi, and Korsmeyer–Peppas to ascertain the drug release mechanism [33].

### 2.10. Quantification of ATZ by HPLC

As per the formerly published method by Konidala et al., a reverse phase high performance liquid chromatographic method (HPLC) with the UV detector method was utilized for the estimation of ATZ in in vitro and in vivo studies. The chromatography was performed utilizing C18 column with specifications of 250 mm × 4.6 mm × 5 µm at 255 nm. Class VP software was utilized for the area calculation of the chromatograms. The method was validated and found to be rapid, simple, sensitive, precise, and accurate. The mobile phase comprised of water: acetonitrile (20:80 *v/v*) with pH adjusted to 3.0 with glacial acetic acid. The mobile phase was propelled at a flow rate of 1 mL/min. Calibration curve was plotted ranging from 100 to 1000 ng/mL. The retention time for ATZ was obtained at 3.9 min. The detection and quantification limit were assessed to be 67.16 ng/mL and 203.53 ng/mL [34].

### 2.11. Ex Vivo Permeation Study

The study was carried out using small intestine isolated from the Wistar rats, which were fasted overnight and sacrificed using the CO_2_ inhalation technique. The intestinal segments were surgically isolated and washed with the Tyrode solution (composed of 15 mM glucose, 136.9 mM sodium chloride, 11.90 mM sodium bicarbonate, 4.2 mM sodium dihydrogen phosphate, 2.7 mM potassium chloride, 1.2 mM calcium chloride, and 0.5 mM magnesium chloride). They were then into segments each measuring 6–7 cm. One end of each segment was tied to form a sac, filled with 1 mL each of NLC and drug suspension and then ligated at the other end. The sacs were suspended for 2 h in 100 mL of Tyrode solution preheated at a temperature of 37 ± 0.5 °C and provided with continuous aeration. Aliquots of 1 mL from each beaker were drawn at preset time-points and replenished with the equal volume of preheated Tyrode solution. The extent of drug permeated beyond the intestinal barrier was estimated by using HPLC [35].

### 2.12. Confocal Laser Scanning Microscopy (CLSM)

This experiment was to assess whether ATZ-NLC crossed BBB and accumulated in the brain upon oral administration. The NLC formulation and drug suspension were treated with rhodamine 123 and administered to the rats via oral feeding sonde. The labeling with the dye was done at the time of the formulation preparation by adding the dye to the melted lipid phase. The rats were sacrificed via the CO_2_ inhalation method and their brains were isolated and washed with PBS pH 7.4. The brain samples were excised into thin sections and examined using CLSM (TCS SP5II, Leica Microsystem Ltd., Wetzlar, Germany). The fluorescence signal was perceived at several depths of the brain indicating the depth of penetration of the formulation [1].

### 2.13. In Vitro Cell Viability Study

The study was executed in Neuro-2a (ATCC CCL-131) brain-derived neuroblastoma cells to assess the cytotoxic activity. About 1 × 10^4^ cells were plated in each well of a 96-well plate for 24 h with Dulbecco’s modified Eagle’s medium (DMEM) accompanied with 10% fetal bovine serum, 100 μg/mL streptomycin, 100 μg/mL penicillin, and 2 mmol/l L-glutamine. Incubation of the cells was done at atmospheric conditions of 37 °C, 100% relative humidity with 95% O_2_/5% CO_2_. The cells were treated for 18 h with samples (ATZ suspension, ATZ-NLC, placebo NLC) of varying concentrations according to the C_max_ of the drug. Thereafter, 10% *w/v* methylthiazoletetrazolium (MTT; 5 mg/mL) was used for the cell treatment, which were then incubated for 4 h at 37 °C. Lastly, the treatment of the cells was done with 10% *w/v* dimethylsulfoxide so that the formazan crystals can be solubilized. The plates were then read using scanning multiwall spectrophotometer and absorbance was recoded at 570 nm [36]. The % cell viability was computed as per the following Equation (4):

(4) $$\%\;Cell\;viability\;=\;\frac{Absorbance\;of\;treated\;cells}{Absorbance\;of\;untreated\;cells}\;\times \;100$$

### 2.14. Histopathological Examination

The brain was isolated from the rats administered with ATZ-suspension and ATZ-NLC. The brain tissues were fixed for 24 h in 10% neutral buffered formalin, which were then washed with water and dehydration by alcohol. The samples were cleaned up using xylene and embedded in paraffin bees wax blocks for an additional 24 h at 56 °C. Slices of thickness 5 μm were cut transversally by a slide microtone and stained. The slices were isolated on glass slides, deparaffinized and then stained with hematoxylin and eosin (H&E) staining and cresyl violet (CV) staining dyes. The brain sections of hippocampus, cortex, and striatum were examined for any histological alterations using the fluorescence motic microscope (Motic AE31) enabled with infinity analyze software [4].

### 2.15. In Vivo Study

For the conduction of the pharmacokinetic studies, the rats were distributed into three groups namely the (1) control group, (2) group administered with ATZ suspension, and (3) group receiving ATZ-NLC with each group containing 15 animals. Using the 18-gauge oral feeding needle, group 1 received saline while group second and third received drug suspension and NLC formulation respectively at the required animal dose of 10.27 mg/kg body weight. The rats were sacrificed using the CO_2_ inhalation method at designated time-points (0.5, 1, 2, 4, 8, and 24) and blood and brain were isolated.

#### 2.15.1. Isolation and Extraction of Plasma and Brain Samples

The blood specimens at each time-point were collected in tubes containing the EDTA anticoagulant. These specimens were then centrifuged at 5000 rpm for 15 min to collect the plasma, which was kept at −20 °C until the day of evaluation. From each sample, 500 μL of plasma was withdrawn and 500 μL of ethyl acetate was added and further centrifuged at 10,000 rpm for 10 min. The supernatant was isolated from each sample in fresh eppendorfs and evaporated to dryness. The residue left was reconstituted with the mobile phase (100 μL), from which 20 μL was injected into the HPLC column [37].

For the estimation of drug content in the rat brain at each time-point, the whole brain from each rat was isolated, washed with isotonic PBS pH 7.4, and thawed until analysis. Thereafter, 1 g of brain was weighed from each sample and 2 mL of PBS pH 7.4 was put in each vial followed by their homogenization. The samples were then extracted with ethyl acetate, vortexed, and centrifuged at 5000 rpm for 15 min. The supernatants were collected, and the samples were extracted again. The supernatants were united and later evaporated to dryness. The residue was reconstituted with 200 μL of mobile phase and filtered with a 0.45 μm nylon membrane filter. Of the filtered sample 20 μL was then injected into HPLC for analysis [38].

#### 2.15.2. Data Analysis

The various pharmacokinetic parameters were computed using the using pharmacokinetic software (PK Functions for Microsoft Excel, Pharsight Corporation, Mountain View, CA, USA). The maximum plasma concentration of ATZ (C_max_) and the time required to achieve the maximum concentration (T_max_) were noted from the actual plasma profiles.

### 2.16. Stability Studies

The formulation ATZ-NLC was preserved in a glass vial and stored for three months at the room temperature (25 ± 2 °C/60% ± 5% RH). The formulation was analyzed at specific time-points of 0, 30, 60, and 90 days for any formation of precipitate, change in physical appearance, particle size, polydispersity index (PDI), and drug content [33].

### 2.17. Statistical Analysis

The outcomes of all studies were replicated thrice and expressed as mean ± SD. The results were compared and examined using a one-way analysis of variance (ANOVA) using Graph Pad Instat 3 (GraphPad InStat Software, Inc., San Diego, CA, USA). The findings with a statistical difference of *p* < 0.05 were deemed to be significant.

## 3. Result and Discussion

### 3.1. Screening of Lipids and Surfactants

The development of NLC formulation with enhanced brain permeability commences with the careful selection of appropriate lipids (solid and liquid) and surfactant. The determination of the drug solubility in different lipids is quintessential as it invariably influences the particle size, EE and LC [39]. All the lipids and surfactants screened for this study belonged to the generally recognized as safe (GRAS) category.

**Figure 2:**
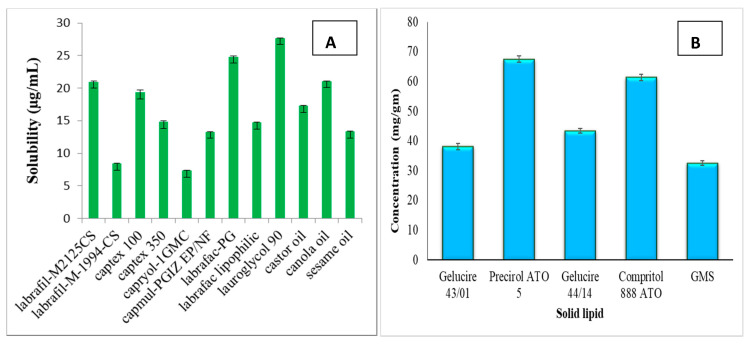
Solubility of atazanavir in (**A**) liquid lipids and (**B**) solid lipids.

Amongst various liquid lipids, ATZ exhibited maximum solubility in Lauroglycol^TM^ 90 (27.69 ± 0.016 mg/mL; Figure 2A). The hydroxyl groups present in the structure of ATZ and the lipid leads to hydrogen bond formation thus enhancing the solubility in addition to the formation of a stable complex [39]. The solid lipid selected for preparing NLC was Precirol^®^ ATO 5 based on its highest ATZ solubilization (67.53 ± 1.027 mg/g; Figure 2B). Precirol^®^ ATO 5 is a blend of diacylglycerols and triacylglycerol, containing short-chain length acylglycerols of distearate, tristearin, and tripalmitin [40]. It is well known that lipids that are a blend of mono-, di-, and triglycerides form crystals with imperfect lattice arrangement thereby offering more space for incorporating drug. Thus, the chemical nature of lipids is another crucial aspect that requires attention, as lipids (monoglycerides) forming highly crystalline particles with an ideal lattice arrangement often leads to drug expulsion [10].

Further, 7:3 was found to be the optimum solid lipid to liquid lipid ratio, at this ratio the lipids were completely miscible exhibiting no indications of phase separation until 72 h of observation and later. The other ratios (60:40, 80:20, and 90:10) showed phase separation after 48 h. The ratio was selected considering the high drug loading capacity in addition to maintaining solid/semisolid texture at room temperature. The high ratio of liquid lipid also ensures high drug encapsulation owing to the high drug solubilization in liquid lipid. It also steers decline in viscosity and surface tension producing particles of small size [41]. Moreover, increasing the concentration of solid lipid leads to the development of perfect crystalline lattice, which reduces the drug entrapment and causes drug expulsion during storage [42].

The emulsification capability of the surfactants was considered as the criteria of their selection for the NLC preparation. Among all the surfactants, Cremophor RH 40 was selected as it showed the highest percentage transmittance of 97.71 (Table 3), which corresponds to smaller sized particles [41]. Moreover, since it is a non-ionic surfactant, it has very low toxic potential, provides rapid brain uptake, and produces smaller particles with narrow particle size distribution [43]. Cremophor RH 40 also possesses additional benefits of P-gp modulation thus inhibiting the efflux of ATZ, which is a P-gp substrate drug. Additionally, the high hydrophilic–lipophilic balance (HLB) value (14-16) of Cremophor RH 40 lowers the interfacial tension present amidst the lipid and aqueous phase thereby imparting stability. The dense hydrophobic tail present in the structure of surfactants and their negative zeta potential deters the aggregation of lipid particles thus imparting steric stabilization to the formulation [40].

### 3.2. Preparation and Optimization of ATZ-NLC

The major motive was to formulate ATZ-NLC with enhanced lymphatic uptake and improvement in oral bioavailability. Therefore the QbD-based approach was utilized to attain a stable formulation showing maximum drug release in a controlled manner when administered orally. The CQAs when kept in limits are critical for attaining the formulation with desired characteristics.

NLC formulations were formulated by melt emulsification followed by the probe sonication method using Precirol^®^ ATO 5 (solid lipid) and Lauroglycol^TM^ 90 (liquid lipid) in the ratio of 70:30 and Cremophor RH 40 (surfactant). 3-factor 3-level BBD when applied, resulted in 17 experimental trials, which are well depicted in Table 4 along with the observed responses.

The design expert software suggested quadratic model for all the responses when they were fitted to the experimental runs. The coefficient of correlation (R^2^) was found to be nearly equal to 1. The positive/negative coefficient in each equation corresponded to the direct/inverse relationship among the different variables. The quadratic equations for the responses, including only significant terms (*p* < 0.05) are as mentioned below:

(5) $$\begin{array}{ll} Particle\;size\;= & +239.46+15.78A-24.01B-55.41C-3.58AB+11.62AC\\ & -12.80BC-23.01A^{2}-2.38B^{2}+46.52C^{2} \end{array}$$

(6) $$\begin{array}{ll} Entrapment\;efficiency\;= & +73.38-2.27A+4.59B+6.29C+1.00AB-2.19AC+2.97BC\\ & +4.73A^{2}+1.31B^{2}-4.12C^{2} \end{array}$$

(7) $$\begin{array}{ll} Drug\;loading= & +6.94-0.1937A+0.3438B+0.6200C+0.0825AB-0.0950AC\\ & +0.1400BC+0.3552A^{2}+0.0502B^{2}-0.4772C^{2} \end{array}$$

where *A* = total lipid concentration (% *w*/*v*), *B* = surfactant concentration (% *w*/*v*), and *C* = sonication time.

#### 3.2.1. Fitting of Data to the Design

The responses on fitting to the experimental design, denoted a good fit. For Equation (5), the value of R^2^, predicted R^2^, and adjusted R^2^ were found to be 0.9807, 0.7608, and 0.9559 respectively. Similarly, for Equation (6), the values were 0.9681, 0.7801, and 0.9271 and for Equation (7), they were 0.9563, 0.7807, and 0.9002 respectively. The values of predicted R^2^ and adjusted R^2^ corresponded to a rational agreement between the two. The adequate precision for Equations (5)–(7) were found to be 20.38, 15.69, and 14.04 respectively, which were greater than 4 (desirable) and thus showed that the signal was adequate.

#### 3.2.2. Response Analysis

Effect of independent variables on the particle size: The particle size of the NLCs varied from 167.1 to 349.2 nm. As according to Equation (5), it was found that the increase in particle size corresponded to the rise in the concentration of the lipid component. This can be attributed to the particle agglomeration occurring as a result of inadequate surfactant concentration required to emulsify the increased lipid phase [29]. However, the surfactant had a negative impact on the particle size, i.e., with the rise in the surfactant concentration, the particle size reduced. This is owing to the diminution in the interfacial tension amidst the lipid phase and aqueous phase, steric stabilization of the particles, and preventing their coalescence to form bigger particles [44]. However, the particle size reduces only up to an optimum surfactant concentration after which it starts increasing due to the deposition of surfactants on the lipid matrix surface [33]. Further, multiplying the sonication time reduced the particle size owing to the breaking of the particles into smaller ones by the high kinetic energy emitted by the probe sonicator [4].

Effect of independent variables on entrapment efficiency and drug loading: Both these parameters, i.e., entrapment efficiency and drug loading were interlinked to each other thus showing similar responses to the variations in independent variables. The %EE was found to vary from 63.66% to 86.75%, while % DL ranged between 5.71% and 7.9%. Equations (6) and (7) clearly depicted the inverse relationship between the lipid concentration and EE or DL. This can be elucidated by the fact that the increase in lipid concentration also resulted in a simultaneous increase in liquid lipid fraction, which cannot be accommodated in the solid lipid matrix. This will result in drug expulsion and thereby decreases % EE and % DL [4]. Contrariwise, with the increase in surfactant concentration, both % EE and % DL were found to increase significantly, which is due to the development of numerous layers around the particle thereby allowing enhanced drug accommodation and retention within the matrix. Moreover, increased surfactant concentration reduced the diffusion speed of the drug by enhancing the viscosity of the aqueous phase consequently leading to enhanced EE and DL [44,45]. Further, increase in sonication times breaks down the bigger particles into smaller particles thus resulting in more drug entrapment thereby increasing % EE and % DL [46].

#### 3.2.3. Contour Plots

**Figure 3:**
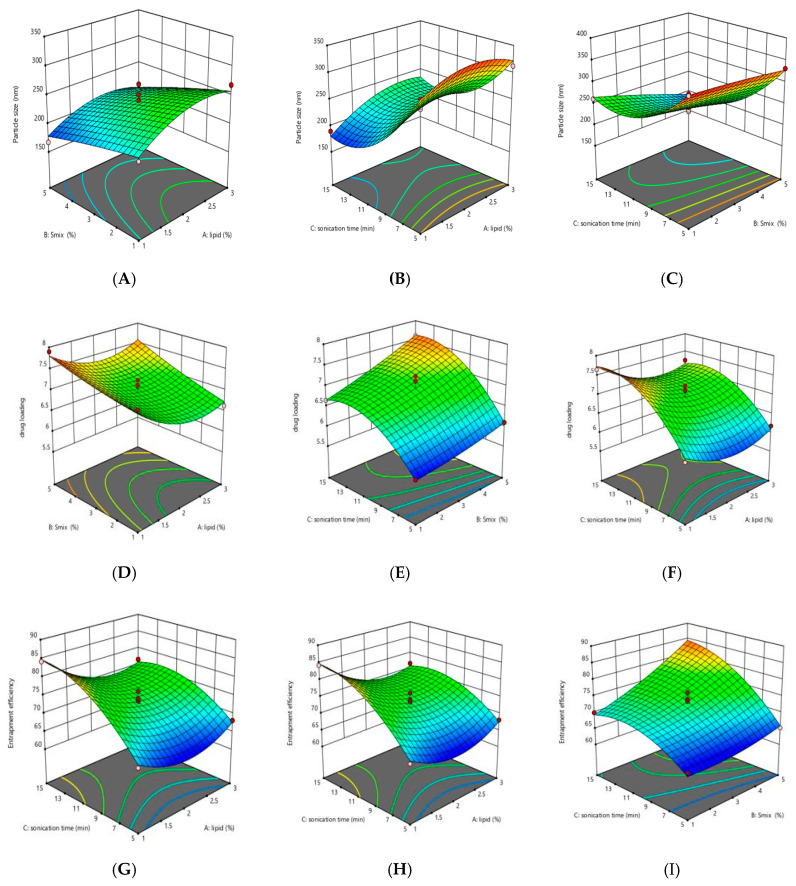
Contour plots representing interaction of (**A**) surfactant and lipid on particle size, (**B**) sonication time and lipid on particle size, (**C**) sonication time and surfactant on particle size, (**D**) surfactant and lipid on %entrapment efficiency, (**E**) sonication time and lipid on %entrapment efficiency, (**F**) sonication time and surfactant %entrapment efficiency, (**G**) surfactant and lipid on %drug loading, (**H**) sonication time and lipid on %drug loading, and (**I**) sonication time and surfactant on %drug loading.

The contour plots for the respective factors and their responses are also shown in Figure 3. These three-dimensional graphs represent the effect of the different independent variables viz. total lipid concentration, surfactant concentration, sonication time on the dependent variables viz. particle size, % entrapment efficiency, and % drug loading.

#### 3.2.4. Validation of Experimental Design

BBD generated the predicted responses of the optimum NLC formulation, which were compared with the obtained responses. The optimized formula obtained by the design included 2% total lipid concentration, 3% surfactant concentration, and 10 min sonication time. The predicted responses for particle size, % EE, and % DL were found to be 239.46 nm, 73.38%, and 6.94%. However, the responses obtained for the formulation prepared using this formula were 227.6 nm, 71.09%, and 8.12% respectively. The results suggested that both the responses were closely correlated thereby establishing the validity and reliability of the optimization method in predicting the optimum NLC formulation.

### 3.3. Evaluation of Optimized ATZ-NLC

#### 3.3.1. Particle Size, PDI, and ZP

**Figure 4:**
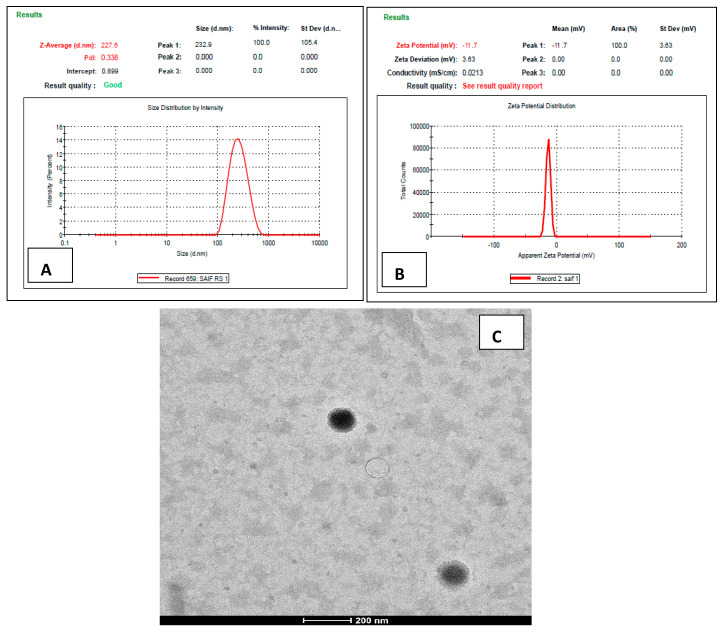
Image representing (**A**) particle size, (**B**) zeta potential, and (**C**) Transmission electron microscopy (TEM) image of optimized ATZ-NLC.

The mean diameter and PDI of the optimized ATZ-NLC were estimated to be 227.6 ± 5.4 nm and 0.338 ± 0.021 respectively as shown in Figure 4A. The particle size was observed to be in the colloidal nanometer range of <550 nm and thus is suitable for brain targeted delivery. The low PDI value indicated uniformity in particle size, higher homogeneity among the particles, and narrow size distribution. The increased particle homogeneity is attributable to the higher interfacial tension amidst aqueous and lipid phase [45]. ZP of the NLC, which was measured to be −11.7 ± 0.47 mV as depicted in Figure 4B. The small ZP could be due to the presence of non-ionic surfactant rendering low magnitude of charge to the nanoparticles [32]. ZP is a significant factor for assessing the stability of the colloidal formulations such as NLC, with values ranging from +30 to −30 mV considered stable [47]. The electrostatic repulsion amidst the particles bearing high positive or negative charge prevents particle aggregation, thus conforming stability to the formulation [48]. Pokharkar and associates also prepared NLC of efavirenz using the melt emulsification–ultrasonication method. The prepared formulations showed the particle size ranging from 150 ± 3.8 to 247 ± 4.1 nm and PDI between 0.1 and 0.3. The zeta potential of all the NLCs varied between −5.58 ± 1.47 and −18.7 ± 1.0 mV [4]

#### 3.3.2. Drug Entrapment Efficiency (% EE) and Drug Loading Capacity (% LC)

ATZ-NLC when characterized for these parameters yielded 71.09% ± 5.84% EE and 8.12% ± 2.7% LC respectively. The high value might be due to the lipophilicity of ATZ, due to which it gets easily solubilized inside the lipid matrix. Moreover, the liquid lipid initiated imperfections in the crystal structure, which allowed more drug to accommodate and did not allow drug to escape to the external medium. Elmowafy and coworkers prepared NLC of atorvastatin, which displayed EE varying from 76% ± 12.4% and 96.6% ± 7.1%. This was owed to the full solubilization of atorvastatin in Capryol PGMC, which produced massive crystal order defects thereby creating space for more drug entrapment [28].

#### 3.3.3. Transmission Electron Microscopy (TEM)

The average diameter of the nanoparticles was found to be within a range of 200 nm as depicted in the Figure 4C. The TEM image demonstrated that the particles were of uniform and spherical shape, without any aggregation. This indicated that the particles in NLC were homogenously dispersed. The results were found to be in conformity with the results of the zetasizer. Similar results were reported by Alam and coworkers where TEM analysis of lamotrigine NLCs revealed spherical particles with uniform size distribution [49].

#### 3.3.4. Differential Scanning Calorimetry (DSC)

**Figure 5:**
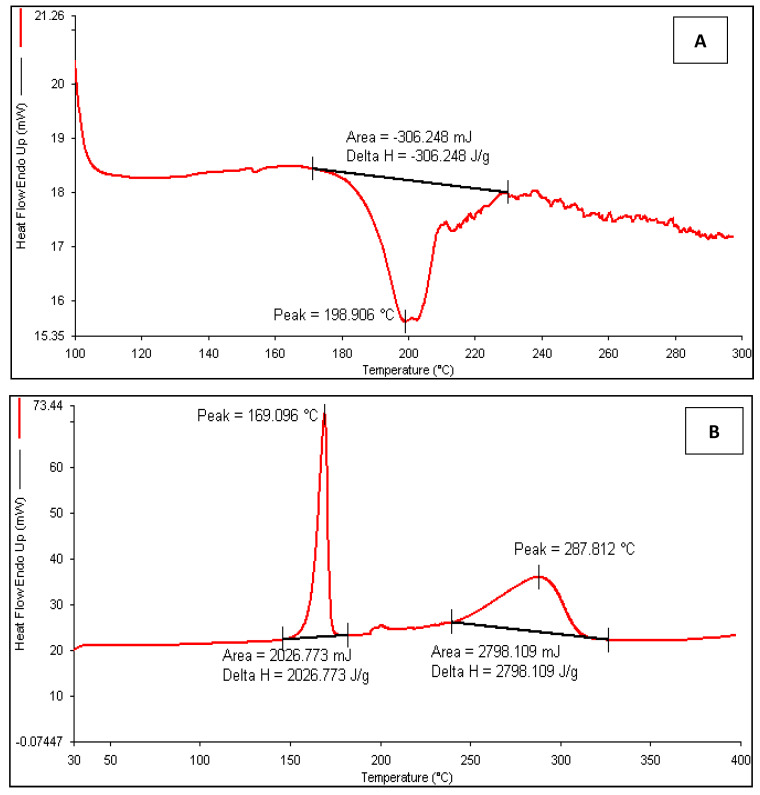
Differential scanning calorimetry (DSC) thermogram of (**A**) ATZ and (**B**) ATZ-NLC.

The DSC thermogram of ATZ in Figure 5 exhibited an exothermic peak at 198.9 °C, which confirms the crystalline nature of the drug. The thermogram of NLC exhibited peaks at 169.1 °C and 287.8 °C, of which the former was equivalent to the melting point of mannitol (cryoprotectant) and the latter might be due to possible impact on crystal lattice of solid lipid by the presence of liquid lipid and surfactant. The results clearly depicted shifting and appearance of a new broad peak, which is indicative of the interaction and encapsulation of the drug inside the lipid matrix. The peak shifting also occurs as the different nanoparticles melt at different temperatures [28,50]. The results were in corroboration with the earlier research, wherein the thermogram of asenapine NLC displayed a broad asymmetric melting peak corresponding to the [40]. Moreover, the lack of characteristic ATZ peak in the NLC thermogram confirms the existence of the drug in the amorphous form or it is dispersed molecularly in the lipid matrix [51].

### 3.4. In Vitro Release Study

**Figure 6:**
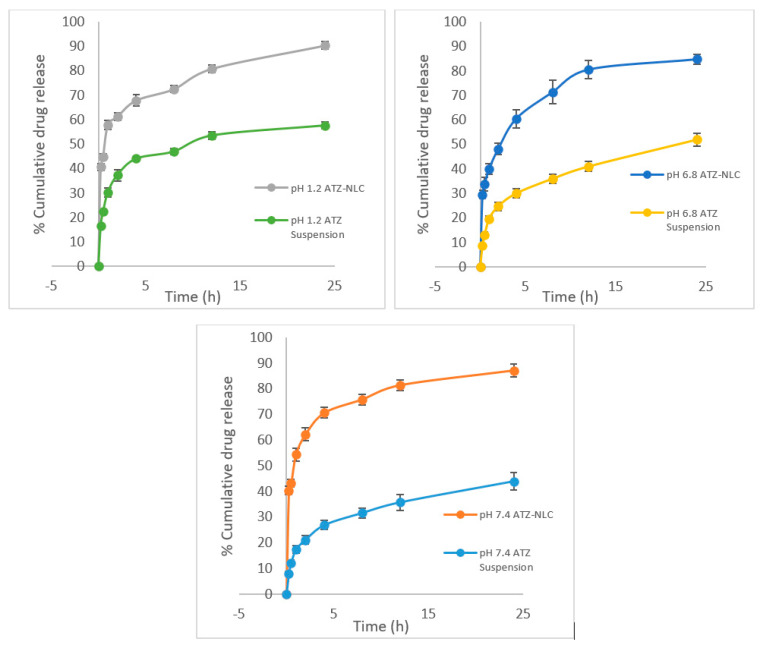
In vitro release profile of ATZ-NLC and ATZ suspension in different buffer media.

The drug release profiles of ATZ from its NLC and suspension are well shown in Figure 6. The profiles of the NLC unveiled a biphasic release showing at first an initial burst release and then trailed by a sustained release for 24 h. The graph shows that more than half of the drug (60%) was released in the first two hours as compared to 30% drug release shown by the drug suspension. The initial burst release could possibly be owed to the drug entrapped on the outer side of NLC. However, the drug entrapped in the solid lipid core was responsible for the sustained release pattern. The diffusion of the aqueous medium inside the core followed by its erosion and drug dissolution leading to the release of the drug. This pattern of biphasic release is favorable for maintaining the drug plasma levels of the patients without causing any fluctuations. The results were in conformity with the earlier research conducted by Seyfoddin and coworkers who prepared NLC of acyclovir. The in vitro release studies of the prepared NLC in PBS pH 7.4 revealed that nearly 60% of the drug was released in less than 3 h from the start of the experiment, which went up to about 90% in 8 h. The reason behind such results was cited by them to be porous nature of NLC, enhanced wettability, and due to the NLC following diffusion and erosion release mechanism [52].

The results revealed that after 24 h, the ATZ release in HCl pH 1.2, PBS pH 6.8, and PBS pH 7.4 was estimated to be 90.38% ± 0.46%, 84.79% ± 2.06%, and 87.11% ± 2.59%. However, for the drug suspension, the drug release in these media was found to be 57.61% ± 1.42%, 51.99% ± 1.04%, and 43.83% ± 0.47% respectively. It is evident that the drug release was highest in the acidic medium, which was because ATZ acts as a mild base exhibiting the highest solubility in the acidic media. Additionally, NLC exhibiting significantly (*p* < 0.001) higher drug release as compared to the suspension might be due to the nanosized particles bearing a large surface area and high drug solubilization in the liquid lipids [53].

The results of the release studies were then fitted to the various kinetic model fitting equations to identify the release kinetics. Based on the R^2^ value (0.974, 0.989, and 0.975), it was discovered that the drug release followed the Korsmeyer–Peppas model with the Fickian diffusion mechanism [54].

### 3.5. Ex Vivo Permeation Study

The HPLC method was validated in Tyrode buffer for all the parameters viz. linearity, precision (interday and intraday), accuracy, and robustness. The run time and retention time was estimated to be 3.90 min and 7 min. The limit of detection and the limit of quantification were computed as 81.46 ng/mL and 246.85 ng/mL. In all the cases the relative standard deviation was within the permissible limit of 2%.

**Figure 7:**
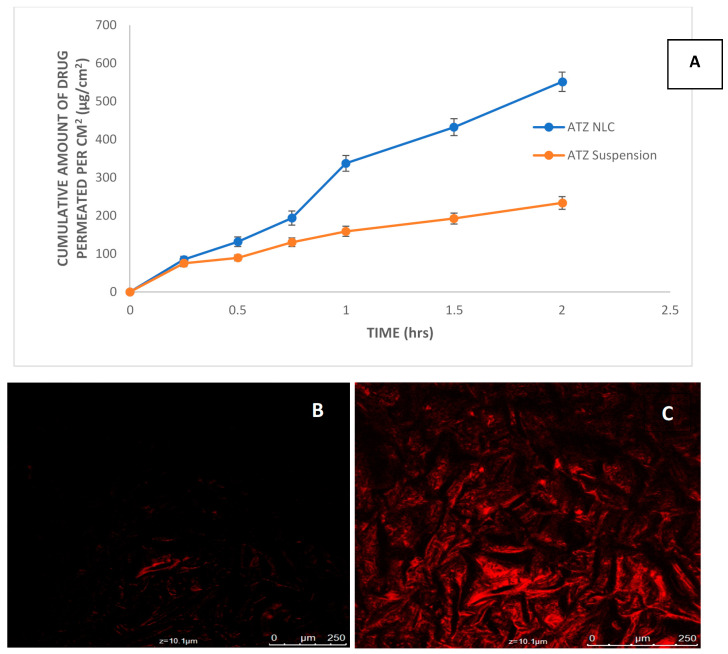
(**A**) Concentration of drug permeated across intestine at different time points. (**B**) Confocal microscopy images of rat brain 2 h after administration of atazanavir suspension. (**C**) Atazanavir NLC.

The intestinal permeability of drug from ATZ-suspension and ATZ-NLC was very well depicted in Figure 7A. The findings indicated that after 2 h, the cumulative amount of drug permeated through rat intestine from NLC (551.4 ± 2.6 μg/cm^2^) was found to be 2.36-folds greater than the drug suspension (233.7 ± 6.1 μg/cm^2^) respectively. The difference in the permeability from both were noticed to be statistically significant at *p* < 0.05. In NLC, the inclusion of lipids and surfactants bearing high permeation potential might be responsible for the enhanced drug permeation across the intestine. The small particle size of NLC was another contributory factor to increased drug permeation. In addition, the surfactants enabled improved partitioning of the drug in the aqueous phase, which necessitated enhanced drug release consequently leading to improved drug permeation. Moreover, the P-gp inhibitory action of Cremophor RH 40 and Lauroglycol 90 mitigated the intestinal efflux of ATZ by impeding the P-gp efflux transporters present in the enterocytes. This also makes the NLC readily available for lymphatic uptake. This study is a good indication of an improved oral bioavailability of ATZ when administered in the form of NLC [36,55]. The results were found to be consistent to the prior research reporting permeation enhancement potential of decitabine-NLC over its suspension by 4-folds. The reason was accredited to the small particle size and the mitigation of the P-gp efflux by the presence of the surfactants [36].

### 3.6. Confocal Laser Scanning Microscopy

Rhodamine 123 is an established biological tracer, and its release is evaluated with respect to the extent of penetration. Being lipophilic, the dye crosses the membranes easily and accumulates in regions with negative membrane potential. Further, the various advantages of this dye like non-invasive detection, no interaction with the formulation components, high quantum yield, and negligible intervention in the underlying metabolic process makes it a versatile biological tracer for microscopical analysis [56,57]. The study was conducted to assess the trafficking and accumulation of NLC in the rat brain after its administration via the oral route. The confocal microscopy images of the rat brain following 2 h of treatment with NLC and drug suspension are shown in Figure 7B,C. These images represent the depth of penetration measured through Z-axis by the presence of fluorescence. At the depth of penetration of 10.1 μm, the images clearly revealed enhanced fluorescence in the case of ATZ-NLC as comparison to ATZ-suspension. The enhanced fluorescence indicated a higher penetration of ATZ-NLC in the brain, attributable to the small nanosized particles and due to the lipids and surfactants, which act as penetration enhancers. Accordingly, it affirmed that NLC could easily cross BBB and accumulated in brain tissues, thus proving its potential in the site-specific drug delivery in neuroAIDS. Chakraborty and coworkers prepared zidovudine NLC and the confocal images of the rat brain revealed that maximum brain accumulation of the NLCs were seen after 4 h of administration, after which the fluorescence was found to decrease [1].

### 3.7. In Vitro Cell Viability Study

**Figure 8:**
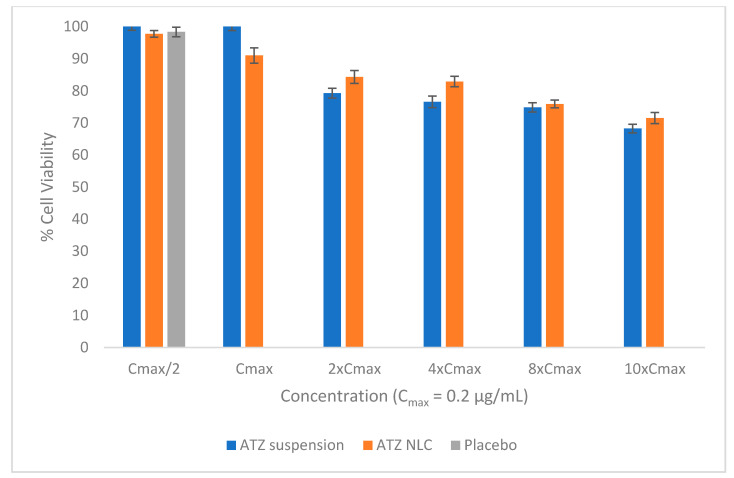
% Cell viability at different C_max_ levels.

The viability of Neuro-2a (ATCC CCL-131) cells was investigated in the presence of drug suspension, placebo NLC, and ATZ-NLC. The study was done at the concentrations ranging from 0.1 to 2 μg/mL, covering the reported C_max_ value of 0.2 μg/mL. The consequence of increasing ATZ concentration on cell viability is depicted in Figure 8. The results revealed dose-dependent cytotoxicity, i.e., the % cell viability for drug suspension and ATZ-NLC samples declined with the increase in the concentration of ATZ from 0.1 to 2 μg/mL. It suggested that the uptake of the ATZ-NLC is concentration-dependent. Moreover, ATZ-NLC and ATZ suspension depicted high % cell viability ranging from 71% to 97% and 68% to 100% at each drug concentration. This indicates that the proposed drug delivery system, i.e., NLC pose low cytotoxicity and is safe to use [42,48]. Further, since the lipid excipients used in formulating NLC/placebo belong to the GRAS category, the placebo did not exhibit any cytotoxicity and its cell viability was found to be 98.33% ± 1.48%. Similar results were reported by Cavalcanti and associates where the lamivudine-loaded NLC displayed less cytotoxicity [48].

### 3.8. Histopathological Examination

**Figure 9:**
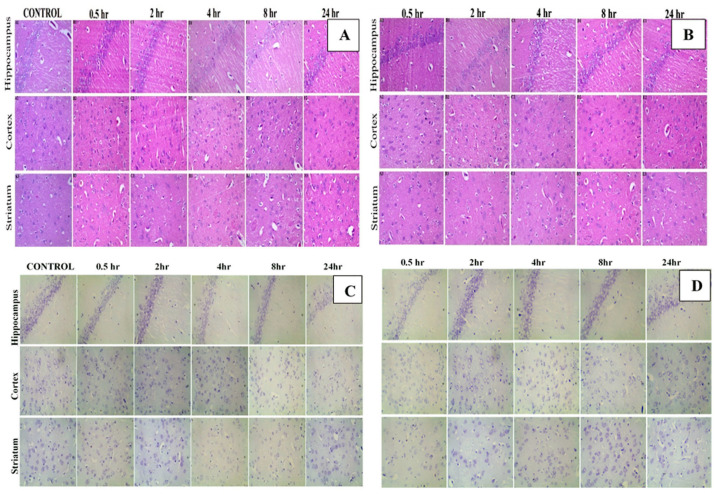
Hematoxylin and eosin staining of the brain for (**A**) ATZ-NLC and (**B**) ATZ suspension. Cresyl violet staining of brain for (**C**) ATZ-NLC and (**D**) ATZ suspension.

The study was carried out to assess the safety of ATZ-NLC and to investigate any possible toxicological or structural aberrations in the brain due to its administration. H&E staining and CV staining of the brain (hippocampus, cortex, and striatum) were done for both ATZ-NLC and ATZ-suspension. The toxicity in the brain after can be estimated by the deep red color of the neurons whereas in CV staining, the neurons take up a deep violet color to exhibit the signs of toxicity. However, the histological findings with H&E staining for ATZ-NLC demonstrated healthy neurons, glial cells, and oligodendrocytes in the hippocampus, cortex, and striatum of the brain, which was confirmed by the light red color neurons with a prominent visible nucleus as depicted in Figure 9A. The results were further validated with the help of CV staining, which depicted light purple colored neurons with prominent centric nucleus as shown in Figure 9C. Additionally, chromatolysis, pyknosis, vacuolization, or neuronal damage in any region of the brain was not observed. The ATZ-suspension also exhibited similar results with no signs of toxicity for both the staining techniques as shown in Figure 9B,D. Thus, based on the histological analysis, it can be concluded that ATZ-NLC did not show any signs of toxicity and is thus safe for oral administration. Rahman and coworkers conducted histopathological studies for Zerumbone-loaded NLC, and the images showed no degenerative, vacuolar, or hemorrhagic alterations in the brain tissues [58].

### 3.9. In Vivo Study

The HPLC method was validated for all the parameters viz. linearity, precision (interday and intraday), accuracy, and robustness. The run time for all the samples was set to 7 min. The retention time in plasma samples and brain samples were estimated to be 3.892 min and 3.925 min respectively. The limit of detection in plasma samples and brain samples were calculated to be 67.16 ng/mL and 65.53 ng/mL and the limit of quantification for the same were calculated as 203.53 ng/mL and 202.64 ng/mL. In all the cases the relative standard deviation was within the permissible limit of 2%.

**Figure 10:**
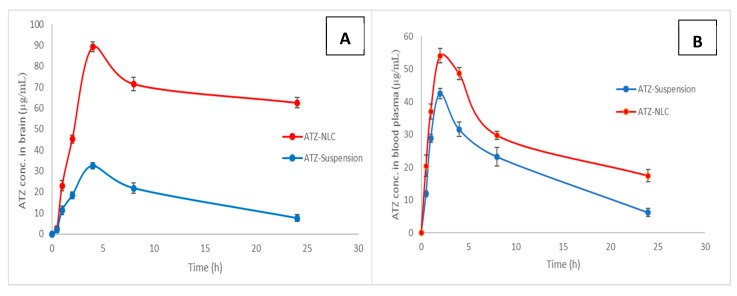
ATZ concentration in (**A**) brain and (**B**) blood plasma.

The brain drug concentration–time profile of ATZ-suspension and ATZ-NLC after oral administration are illustrated in Figure 10A. It was observed that at each timepoint, the ATZ concentration in CNS was found to be more in the groups receiving NLC as compared to those administered with drug suspension. The difference in the CNS concentration in both the groups was found to be significant (*p* < 0.01). However, there was no difference in T_max_ in both the groups. The highest concentration of ATZ, i.e., C_max_ in brain for ATZ-NLC (89.24 ± 2.23 µg/mL) was found to be 2.75-folds greater than drug suspension (32.46 ± 1.49 µg/mL). The area under the curve (AUC) for NLC was estimated to be 1570.41 ± 112.62 µg.h/mL, which was higher than ATZ-suspension (413.04 ± 31.36 µg.h/mL). Thus, approximately 4-fold improvement in the bioavailability was observed by formulating ATZ-loaded NLC. Meanwhile, the drug concentration in the brain after 24 h of NLC administration was found to be 62.57 ± 2.43 µg/mL, whereas it was only 7.54 ± 1.68 µg/mL in the case of drug suspension. This clearly defined the superiority of NLC over suspension in the delivering drug to the brain [59]. Similarly, as depicted in Figure 10B, the administration of NLC also exhibited higher plasma C_max_ of 54.12 ± 2.17 µg/mL as compared to drug suspension (42.53 ± 1.57 µg/mL) with no difference in T_max_. ATZ-NLC 705.67 ± 64.18 µg.h/mL) depicted a 1.5-fold increase in AUC over drug suspension (468.30 ± 52.93 µg.h/mL), thus indicating higher absorption with NLC formulation. It is noted that at the same time points, the brain concentration of ATZ-NLC was much higher than its plasma concentrations. Khan and associates reported similar results wherein the brain concentration of carbamazepine-loaded NLCs was much higher as compared to the plasma concentrations. The NLCs increased the brain enhancement factor of the drug by 1.35-folds in comparison to the suspension [60].

Further, the plasma and brain concentrations of ATZ from drug suspension were more or less equal, showing no significant difference between the two. The likely explanations of the low oral bioavailability of ATZ include its poor water-solubility, extensive hepatic metabolism by CYP3A, and P-gp mediated intestinal efflux. The low solubility was responsible for most of the drug to exist as undissolved particles leading to low C_max_ and AUC for the drug suspension. However, these problems were potentially overcome by formulating its NLC. This increase in drug concentration in CNS could be attributed to the amorphous ATZ, which is molecularly dispersed in the lipid matrix of NLC. The lipids used in the NLC were physiological lipids, thus mimicking the fat-rich food thereby leading to the secretion of bile in the small intestines. The NLCs combine with bile salts to form micelles, which endure lymphatic uptake consequently avoiding the hepatic metabolism [61,62]. The nanoparticles also protected the drug from enzymatic degradation and reduced the uptake by reticuloendothelial system. Further, the small particle size with large surface area, high dispersibility, and prolonged residence time were also responsible for attaining a steady absorption. The P-gp inhibiting activity of the incorporated lipids and surfactants is yet reason behind improved bioavailability with NLC, as it minimizes the P-gp efflux of ATZ occurring at intestinal and brain endothelial cells [60]. Altogether, these factors consequently contribute to achieving an effective and targeted delivery of ATZ to the brain for an effective management of neuroAIDS.

### 3.10. Stability Studies

The ATZ-NLC kept at the specified conditions for 3 months showed no noticeable change in appearance and color on visual inspection. The formulation was observed to be stable with no precipitate formation, phase separation, and degradation. Additionally, no variation in PDI, particle size, and drug content were observed. The small particle size with high homogeneity and stabilization by the surfactants might be responsible for attaining a stable formulation. The results of the stability studies are depicted in Table 5.

## 4. Conclusions

The present research discussed the successful development of ATZ-loaded NLC using melt emulsification followed by the probe sonication method. Implementation of BBD yielded the formula for an optimized NLC formulation with a small particle size, high entrapment efficiency, and high drug loading. The formulation characterized on various parameters demonstrated a biphasic release pattern, enhanced permeation potential, and was found to be safe, reliable, and lacking any toxicity. The pharmacokinetic study revealed a substantial increase in brain availability of the drug with about 4-fold increment in bioavailability when compared to the drug suspension. Based on the outcomes it might be concluded that NLCs proved to be a versatile drug delivery vehicle for brain targeting and enhancing the biopharmaceutical attributes. It is therefore inevitable that NLCs can be successfully employed in the management of neuroAIDS with a potential to decrease the dosage and dosing frequency thereby enhancing the patient compliance.

## Figures and Tables

**Table 1:** Elements of the quality target product profile (QTPP) and critical quality attribute (CQA) for the development of the atazanavir-nanostructured lipid carrier (ATZ-NLC).

**QTPP**		
**QTPP**	**Target**	**Justification**
Drug delivery system	Nanostructured lipid carriers	Offers augmentation in oral bioavailability
Dosage type	Controlled release	Enhanced drug absorption will be achieved
Route of administration	Oral	Patient compliant route offering lymphatic uptake.
Drug release	More than 80%	Essential for attained optimal therapeutic activity
**CQA**		
**CQA**	**Target**	**Justification**
Particle size	Less than 250 nm	Absorption and bioavailability are improved
Entrapment efficiency	More than 70%	Improved therapeutic outcome and pharmacological activity.
Drug loading	More than 8%	Improved therapeutic outcome and pharmacological activity

**Table 2:** Different levels and goals for the variables in the Box–Behnken design.

Variables	Levels		
**Independent variables**	**Low (−1)**	**Medium (0)**	**High (+1)**
Total Lipid (%)	1	2	3
Surfactant (%)	1	3	5
Sonication time (min)	5	10	15
**Dependent variables**	**Goals**		
Particle size (nm)	Minimize		
Entrapment efficiency (%)	Maximize		
Drug Loading (%)	Maximize		

**Table 3:** % Transmittance of surfactants.

Surfactants	% Transmittance
Span 20	0.744
Poloxamer	2.78
Cremophor RH40	97.71
Poloxamer 188	9.77
Tween 20	45.57
Tween 80	73.15
Solutol HS15	48.5

**Table 4:** Experimental runs designed by Box–Behnken and the obtained responses.

Std	Run	A: Total Lipid %	B: Surfactant %	C: Sonication Time (min)	Particle Size (nm)	Entrapment Efficiency %	Loading Capacity %
4	1	3	5	10	208.4	82.93	7.5
16	2	2	3	10	237.4	70.98	6.85
13	3	2	3	10	231.8	73.43	7.24
17	4	2	3	10	235.4	76.18	7.12
10	5	2	5	5	331.4	65.22	6.1
2	6	3	1	10	268.2	70.08	6.63
8	7	3	3	15	227.8	76.62	7.25
9	8	2	1	5	349.2	63.66	5.71
15	9	2	3	10	241.4	74.12	6.78
3	10	1	5	10	167.1	86.75	7.9
14	11	2	3	10	251.3	72.19	6.72
5	12	1	3	5	321.4	66.98	6.2
12	13	2	5	15	192.4	83.43	7.6
7	14	1	3	15	189.9	84.23	7.65
6	15	3	3	5	312.8	68.12	6.18
11	16	2	1	15	261.4	69.98	6.65
1	17	1	1	10	212.6	77.92	7.36

**Table 5:** Stability of ATZ-NLC for three months storage at 25 ± 2 °C/60% ± 5% RH.

Period (Days)	Particle Size (nm)	PDI	Entrapment Efficiency (%)	Physical Appearance	Phase Separation	Precipitate Formation
0	230.2 ± 4.14	0.342 ± 0.022	71.5 ± 4.13	No change	No	No
30	234.6 ± 5.26	0.357 ± 0.031	71.3 ± 2.48	No change	No	No
60	237.5 ± 4.76	0.401 ± 0.025	70.1 ± 1.46	No change	No	No
90	238.6 ± 5.28	0.418 ± 0.027	68.8 ± 3.29	No change	No	No

## Abbreviations

AIDS	Acquired immunodeficiency syndrome
WHO	World Health Organization
UNAIDS	The Joint United Nations Program on HIV/AIDS
HIV	Human immunodeficiency virus
HAART	Highly active antiretroviral therapy
ARV	Antiretroviral
CNS	Central nervous system
BBB	Blood–brain barrier
P-gp	P-glycoprotein
NLC	Nanostructured lipid carriers
ATZ	Atazanavir
SLN	Solid lipid nanoparticle
SNEDDS	Self-nanoemulsifying drug delivery system
SLB	Solid–liquid binary lipid
QbD	Quality by Design
BBD	Box–Behnken design
RSM	Response surface methodology
PDI	Polydispersity index
ZP	Zeta potential
EE	Entrapment efficiency
LC	Loading capacity
TEM	Transmission electron microscopy
DSC	Differential scanning calorimetry
PBS	Phosphate buffer saline
HPLC	High performance liquid chromatographic method
CLSM	Confocal laser scanning microscopy
MTT	Methylthiazoletetrazolium
H&E	Hematoxylin and eosin
CV	Cresyl violet
ANOVA	Analysis of variance (ANOVA
GRAS	Generally recognized as safe
HLB	Hydrophilic–lipophilic balance

## References

[1] Chakraborty T., Das M.K., Dutta L., Mukherjee B., Das S., Sarma A. (2019). Successful Delivery of Zidovudine-Loaded Docosanol Nanostructured Lipid Carriers (Docosanol NLCs) into Rat Brain. Surface Modification of Nanoparticles for Targeted Drug Delivery.

[2] Unaids.org (2018). Switzerland: UNAIDS. https://www.unaids.org/en/regionscountries/countries/india.

[3] Sarma A., Das M.K. (2019). Formulation by Design (FbD) approach to develop Tenofovir Disoproxil Fumarate loaded Nanostructured Lipid Carriers (NLCs) for the aptness of nose to brain delivery. J. Drug Deliv. Ther..

[4] Pokharkar V., Patil-Gadhe A., Palla P. (2017). Efavirenz loaded nanostructured lipid carrier engineered for brain targeting through intranasal route: In-vivo pharmacokinetic and toxicity study. Biomed. Pharmacother..

[5] Chiappetta D.A., Höcht C., Opezzo J.A.W., Sosnik A. (2013). Intranasal administration of antiretroviral-loaded micelles for anatomical targeting to the brain in HIV. Nanomedicine.

[6] Dalpiaz A., Ferraro L., Perrone D., Leo E., Iannuccelli V., Pavan B., Paganetto G., Beggiato S., Scalia S. (2014). Brain Uptake of a Zidovudine Prodrug after Nasal Administration of Solid Lipid Microparticles. Mol. Pharm..

[7] Nair M., Jayant R.D., Kaushik A., Sagar V. (2016). Getting into the brain: Potential of nanotechnology in the management of NeuroAIDS. Adv. Drug Deliv. Rev..

[8] Raymond A., Diaz P., Chevelon S., Agudelo M., Yndartarias A., Ding H., Kaushik A., Jayant R.D., Nikkhah-Moshaie R., Roy U. (2016). Microglia-derived HIV Nef+ exosome impairment of the blood–brain barrier is treatable by nanomedicine-based delivery of Nef peptides. J. NeuroVirology.

[9] Alex A., Paul W., Chacko A.J., Sharma C.P. (2011). Enhanced delivery of lopinavir to the CNS using Compritol^®^-based solid lipid nanoparticles. Ther. Deliv..

[10] Makwana V., Jain R., Patel K., Nivsarkar M., Joshi A. (2015). Solid lipid nanoparticles (SLN) of Efavirenz as lymph targeting drug delivery system: Elucidation of mechanism of uptake using chylomicron flow blocking approach. Int. J. Pharm..

[11] Das S., Ng W.K., Tan R.B. (2012). Are nanostructured lipid carriers (NLCs) better than solid lipid nanoparticles (SLNs): Development, characterizations and comparative evaluations of clotrimazole-loaded SLNs and NLCs?. Eur. J. Pharm. Sci..

[12] Kasongo K.W., Shegokar R., Müller R.H., Walker R.B. (2010). Formulation development and in vitro evaluation of didanosine-loaded nanostructured lipid carriers for the potential treatment of AIDS dementia complex. Drug Dev. Ind. Pharm..

[13] Annu, Rehman S., Shadab, Baboota S., Ali J. (2019). Analyzing Nanotheraputics-Based Approaches for the Management of Psychotic Disorders. J. Pharm. Sci..

[14] Wagner S., Zensi A., Wien S.L., Tschickardt S.E., Maier W., Vogel T., Worek F., Pietrzik C.U., Kreuter J., Von Briesen H. (2012). Uptake Mechanism of ApoE-Modified Nanoparticles on Brain Capillary Endothelial Cells as a Blood-Brain Barrier Model. PLoS ONE.

[15] Clark A.J., Davis M.E. (2015). Increased brain uptake of targeted nanoparticles by adding an acid-cleavable linkage between transferrin and the nanoparticle core. Proc. Natl. Acad. Sci. USA.

[16] Van Rooy I., Cakir-Tascioglu S., Hennink W.E., Storm G., Schiffelers R.M., Mastrobattista E. (2011). In Vivo Methods to Study Uptake of Nanoparticles into the Brain. Pharm. Res..

[17] Santa-Maria A.R., Walter F.R., Valkai S., Brás A.R., Mészáros M., Kincses A., Klepe A., Gaspar D., Castanho M.A., Zimányi L. (2019). Lidocaine turns the surface charge of biological membranes more positive and changes the permeability of blood-brain barrier culture models. Biochim. Biophys. Acta Biomembr..

[18] Singh G., Pai R.S. (2016). Atazanavir-loaded Eudragit RL 100 nanoparticles to improve oral bioavailability: Optimization and in vitro/in vivo appraisal. Drug Deliv..

[19] Singh G., Pai R.S. (2014). Optimized self-nanoemulsifying drug delivery system of atazanavir with enhanced oral bioavailability:in vitro/in vivocharacterization. Expert Opin. Drug Deliv..

[20] Chattopadhyay N., Zastre J., Wong H.-L., Wu X.Y., Bendayan R. (2008). Solid Lipid Nanoparticles Enhance the Delivery of the HIV Protease Inhibitor, Atazanavir, by a Human Brain Endothelial Cell Line. Pharm. Res..

[21] Müller R., Radtke M., Wissing S. (2002). Solid lipid nanoparticles (SLN) and nanostructured lipid carriers (NLC) in cosmetic and dermatological preparations. Adv. Drug Deliv. Rev..

[22] Chatterjee B., Almurisi S.H., Dukhan A.A.M., Mandal U.K., Sengupta P. (2016). Controversies with self-emulsifying drug delivery system from pharmacokinetic point of view. Drug Deliv..

[23] Gambhire V., Narkhede R., Gujar K. (2014). Design and evaluation of self-nanoemulsifying drug delivery systems for nebivolol hydrochloride. Asian, J. Pharm..

[24] Devkar T.B., Tekade A.R., Khandelwal K.R. (2014). Surface engineered nanostructured lipid carriers for efficient nose to brain delivery of ondansetron HCl using Delonix regia gum as a natural mucoadhesive polymer. Coll. Surf. B Biointerf..

[25] Shete H., Patravale V. (2013). Long chain lipid based tamoxifen NLC. Part I: Preformulation studies, formulation development and physicochemical characterization. Int. J. Pharm..

[26] Iqbal B., Ali J., Baboota S. (2018). Silymarin loaded nanostructured lipid carrier: From design and dermatokinetic study to mechanistic analysis of epidermal drug deposition enhancement. J. Mol. Liq..

[27] Cunha S., Costa C.P., Loureiro J.A., Alves J., Peixoto A.F., Forbes B., Lobo J.M.S., Silva A. (2020). Double Optimization of Rivastigmine-Loaded Nanostructured Lipid Carriers (NLC) for Nose-to-Brain Delivery Using the Quality by Design (QbD) Approach: Formulation Variables and Instrumental Parameters. Pharmaceutics.

[28] Elmowafy M., Ibrahim H.M., Ahmed M.A., Shalaby K., Salama A., Hefesha H. (2017). Atorvastatin-loaded nanostructured lipid carriers (NLCs): Strategy to overcome oral delivery drawbacks. Drug Deliv..

[29] Jazuli I., Nabi B., Moolakkadath T., Alam T., Baboota S., Ali J., Annu, Annu I.J. (2019). Optimization of Nanostructured Lipid Carriers of Lurasidone Hydrochloride Using Box-Behnken Design for Brain Targeting: In Vitro and In Vivo Studies. J. Pharm. Sci..

[30] Kudarha R., Dhas N.L., Pandey A., Belgamwar V.S., Ige P.P. (2014). Box–Behnken study design for optimization of bicalutamide-loaded nanostructured lipid carrier: Stability assessment. Pharm. Dev. Technol..

[31] Tsai M.-J., Wu P.-C., Huang Y.-B., Chang J.-S., Lin C.-L., Tsai Y.-H., Fang J.-Y. (2012). Baicalein loaded in tocol nanostructured lipid carriers (tocol NLCs) for enhanced stability and brain targeting. Int. J. Pharm..

[32] Duong V.-A., Nguyen T.-T.-L., Maeng H.-J., Chi S.-C. (2019). Nanostructured lipid carriers containing ondansetron hydrochloride by cold high-pressure homogenization method: Preparation, characterization, and pharmacokinetic evaluation. J. Drug Deliv. Sci. Technol..

[33] Shah B., Khunt D., Bhatt H., Misra M., Padh H. (2016). Intranasal delivery of venlafaxine loaded nanostructured lipid carrier: Risk assessment and QbD based optimization. J. Drug Deliv. Sci. Technol..

[34] Konidala S.K., Sujana K., Rani A.P. (2012). New Validated RP-HPLC method for the Determination of Atazanavir Sulphate in Bulk and Dosage form. Der. Pharma. Chemica..

[35] Rehman S., Nabi B., Fazil M., Khan S., Bari N.K., Singh R., Ahmad S., Kumar V., Baboota S., Ali J. (2017). Role of P-Glycoprotein Inhibitors in the Bioavailability Enhancement of Solid Dispersion of Darunavir. BioMed Res. Int..

[36] Neupane Y.R., Srivastava M., Ahmad N., Kumar N., Bhatnagar A., Kohli K. (2014). Lipid based nanocarrier system for the potential oral delivery of decitabine: Formulation design, characterization, ex vivo, and in vivo assessment. Int. J. Pharm..

[37] Sharma S., Rabbani S.A., Narang J.K., Pottoo F.H., Ali J., Kumar S., Baboota S. (2020). Role of Rutin Nanoemulsion in Ameliorating Oxidative Stress: Pharmacokinetic and Pharmacodynamics Studies. Chem. Phys. Lipids.

[38] Garg B., Beg S., Kumar R., Katare O., Singh B. (2019). Nanostructured lipidic carriers of lopinavir for effective management of HIV-associated neurocognitive disorder. J. Drug Deliv. Sci. Technol..

[39] Kasongo K.W., Pardeike J., Müller R.H., Walker R.B. (2011). Selection and Characterization of Suitable Lipid Excipients for use in the Manufacture of Didanosine-Loaded Solid Lipid Nanoparticles and Nanostructured Lipid Carriers. J. Pharm. Sci..

[40] Alam T., Khan S., Gaba B., Haider F., Baboota S., Ali J. (2018). Adaptation of Quality by Design-Based Development of Isradipine Nanostructured–Lipid Carrier and Its Evaluation for In Vitro Gut Permeation and In Vivo Solubilization Fate. J. Pharm. Sci..

[41] Negi L.M., Jaggi M., Talegaonkar S. (2014). Development of protocol for screening the formulation components and the assessment of common quality problems of nano-structured lipid carriers. Int. J. Pharm..

[42] Meng F., Asghar S., Xu Y., Wang J., Jin X., Wang Z., Wang J., Ping Q., Zhou J., Xiao Y. (2016). Design and evaluation of lipoprotein resembling curcumin-encapsulated protein-free nanostructured lipid carrier for brain targeting. Int. J. Pharm..

[43] Kreuter J. (2004). Influence of the Surface Properties on Nanoparticle-Mediated Transport of Drugs to the Brain. J. Nanosci. Nanotechnol..

[44] Mandpe L., Pokharkar V. (2013). Quality by design approach to understand the process of optimization of iloperidone nanostructured lipid carriers for oral bioavailability enhancement. Pharm. Dev. Technol..

[45] Fatima N., Rehman S., Nabi B., Baboota S., Ali J. (2019). Harnessing nanotechnology for enhanced topical delivery of clindamycin phosphate. J. Drug Deliv. Sci. Technol..

[46] De Souza I.D.L., Saez V., De Campos V.E.B., Mansur C.R.E. (2019). Size and Vitamin E Release of Nanostructured Lipid Carriers with Different Liquid Lipids, Surfactants and Preparation Methods. Macromol. Symp..

[47] Jia L.-J., Zhang D.-R., Li Z.-Y., Feng F.-F., Wang Y., Dai W.-T., Duan C.-X., Zhang Q. (2009). Preparation and characterization of silybin-loaded nanostructured lipid carriers. Drug Deliv..

[48] Cavalcanti S.M.T., Nunes C., Lima S.A.C., Soares-Sobrinho J.L., Reis S. (2017). Multiple Lipid Nanoparticles (MLN), a New Generation of Lipid Nanoparticles for Drug Delivery Systems: Lamivudine-MLN Experimental Design. Pharm. Res..

[49] Alam T.P.J., Vohora D., Aqil M., Aqil M., Ali A., Sultana Y. (2015). Optimization of nanostructured lipid carriers of lamotrigine for brain delivery: In vitro characterization and in vivo efficacy in epilepsy. Expert Opin. Drug Deliv..

[50] Singh S.K., Dadhania P., Vuddanda P.R., Jain A., Velaga S. (2016). Intranasal delivery of asenapine loaded nanostructured lipid carriers: Formulation, characterization, pharmacokinetic and behavioural assessment. RSC Adv..

[51] Gabal Y.M., Kamel A.O., Sammour O.A., Elshafeey A.H. (2014). Effect of surface charge on the brain delivery of nanostructured lipid carriers in situ gels via the nasal route. Int. J. Pharm..

[52] Seyfoddin A., Al-Kassas R. (2012). Development of solid lipid nanoparticles and nanostructured lipid carriers for improving ocular delivery of acyclovir. Drug Dev. Ind. Pharm..

[53] Muthu M.S., Sahu A.K., Sonali, Abdulla A., Kaklotar D., Rajesh C.V., Singh S., Pandey B.L. (2014). Solubilized delivery of paliperidone palmitate by d-alpha-tocopheryl polyethylene glycol 1000 succinate micelles for improved short-term psychotic management. Drug Deliv..

[54] Jindal A.B., Bachhav S.S., Devarajan P.V. (2017). In situ hybrid nano drug delivery system (IHN-DDS) of antiretroviral drug for simultaneous targeting to multiple viral reservoirs: An in vivo proof of concept. Int. J. Pharm..

[55] Ahmadi N., Rostamizadeh K., Modarresi-Alam A.R. (2018). Therapeutic Anti-Inflammatory Potential of Different Formulations Based on Coenzyme Q10-Loaded Nanostructured Lipid Carrier: In Vitro, Ex vivo, and In Vivo Evaluations. Eur. J. Lipid Sci. Technol..

[56] Nabi B., Rehman S., Aggarwal S., Baboota S., Ali J. (2020). Quality by Design Adapted Chemically Engineered Lipid Architectonics for HIV Therapeutics and Intervention: Contriving of Formulation, Appraising the In vitro Parameters and In vivo Solubilization Potential. AAPS PharmSciTech.

[57] Forster S., Thumser A.E., Hood S.R., Plant N. (2012). Characterization of Rhodamine-123 as a Tracer Dye for Use In In vitro Drug Transport Assays. PLoS ONE.

[58] Rahman H.S., Rasedee A., Othman H.H., Chartrand M.S., Namvar F., Yeap S.K., Samad N.A., Andas R.J., Nadzri N.M., Anasamy T. (2014). Acute Toxicity Study of Zerumbone-Loaded Nanostructured Lipid Carrier on BALB/c Mice Model. BioMed Res. Int..

[59] Singh S.K., Hidau M.K., Gautam S., Gupta K., Singh K.P., Singh S.K. (2018). Glycol chitosan functionalized asenapine nanostructured lipid carriers for targeted brain delivery: Pharmacokinetic and teratogenic assessment. Int. J. Biol. Macromol..

[60] Khan N., Shah F.A., Rana I., Ansari M.M., Din F.U., Rizvi S.Z.H., Aman W., Lee G.-Y., Lee E.-S., Kim J.-K. (2020). Nanostructured lipid carriers-mediated brain delivery of carbamazepine for improved in vivo anticonvulsant and anxiolytic activity. Int. J. Pharm..

[61] Zhuang C.-Y., Li N., Wang M., Zhang X.-N., Pan W., Peng J.-J., Pan Y.-S., Tang X. (2010). Preparation and characterization of vinpocetine loaded nanostructured lipid carriers (NLC) for improved oral bioavailability. Int. J. Pharm..

[62] Misra S., Chopra K., Sinha V.R., Medhi B. (2015). Galantamine-loaded solid–lipid nanoparticles for enhanced brain delivery: Preparation, characterization, in vitro and in vivo evaluations. Drug Deliv..

